# FROM COMFORT TO PROFITABILITY: RELATIONSHIP BETWEEN CARE QUALITY AND FINANCIAL PERFORMANCE IN US HOSPICES

**DOI:** 10.1093/geroni/igad104.1544

**Published:** 2023-12-21

**Authors:** Ganisher Davlyatov, Mengying He, Deepthi Padakandla, Robert Weech-Maldonado

**Affiliations:** University of Oklahoma Health Sciences Center, Oklahoma City, Oklahoma, United States; California State University, Los Angeles, California, United States; University of Oklahoma Health Sciences Center, Oklahoma City, Oklahoma, United States; University of Alabama at Birmingham, Birmingham, Alabama, United States

## Abstract

Hospice care is a critical aspect of end-of-life care in the United States, providing comfort and support to individuals with life-limiting illnesses. Quality of care is a key concern for hospice facilities, as it can impact both patient outcomes and financial sustainability. Poor quality of care can lead to negative patient experiences, increased hospitalizations, and even legal issues. It can also result in financial penalties or reduced reimbursement from payers. However, there is limited research on the relationship between quality of care and financial performance in hospice facilities. Employing a cross-sectional observational design, we examined the relationship between Hospice and Palliative Care Composite Process Measure (lagged by 1 year) and the total margin of hospice facilities across the United States. We used secondary data from Nursing Home Compare, Healthcare Cost Report Information System Dataset, and the American Community Survey for the period 2017-2021. We found a positive association between quality process measures and financial performance in hospice facilities after controlling for organizational and community factors that may influence financial performance, such as facility size and location. Specifically, a 10-unit increase in quality measure was associated with a 2% increase in total margin. The findings of this study have important implications for the management and financing of hospice facilities. Investments in quality improvement initiatives may lead to improved financial performance. Policymakers should consider these findings when designing payment systems for hospice care, and administrators of hospice facilities should prioritize quality improvement initiatives to ensure both high-quality care and financial viability.

